# Metabolic responses to acute sprint interval exercise training performed after an oral 75‐gram glucose load in individuals with overweight/obesity

**DOI:** 10.14814/phy2.15555

**Published:** 2023-01-25

**Authors:** Hugo Alejandro Carrillo‐Arango, Miguel Alejandro Atencio‐Osorio, Carlos Alejandro López‐Álban, Edna J. Nava‐González, María Correa‐Rodríguez, Mikel Izquierdo, Robinson Ramírez‐Vélez

**Affiliations:** ^1^ Grupo de Investigación en Deporte de Rendimiento (GRINDER), Programa de Educación Física y Deporte Universidad del Valle Cali Colombia; ^2^ Zoe H&F Centro Para la Investigación En Salud y Rendimiento Humano Cali Colombia; ^3^ Facultad de Salud Pública y Nutrición Universidad Autónoma de Nuevo León Monterrey, Nuevo León México; ^4^ Department of Nursing, Faculty of Health Sciences University of Granada Granada Spain; ^5^ Biosanitary Research Institute (ibs.GRANADA) Granada Spain; ^6^ Navarrabiomed, Hospital Universitario de Navarra (HUN), Universidad Pública de Navarra (UPNA), Instituto de Investigación Sanitaria de Navarra (IdiSNA) Pamplona España; ^7^ CIBER of Frailty and Healthy Aging (CIBERFES) Instituto de Salud Carlos III Madrid Spain; ^8^ Facultad de Ciencias de la Educación Unidad Central del Valle del Cauca (UCEVA) Túlua Colombia

**Keywords:** acute sprint interval training, carbohydrate substrate utilization, fat substrate utilization, metabolic response, postprandial glucose, respiratory quotient

## Abstract

There is evidence supporting that acute sprint interval training (SIT) might improve metabolic responses to postprandial glucose, but results are inconclusive. The aim of the present study was to explore the effects of acute SIT on metabolic response and substrate utilization in individuals with overweight/obesity after an oral 75‐gram glucose challenge. Thirty‐three participants with overweight/ obesity (32.7 ± 8.3 years, 24 male, 9 female) participated in the study and a crossover design was followed. After the 75‐gram glucose load, participants were randomly allocated to two groups: no exercise (resting) or SIT protocol. Metabolic data including respiratory quotient (RQ) and substrate utilization rates (fats and carbohydrates) were collected using the COSMED Q‐NRG + ® calorimeter. The RQ was significantly lower in the acute SIT group (0.76 [0.01]; *p* < 0.0001) than in the resting group (0.80 [0.01]; *p* = 0.036) at the 120‐min postprandial time point, and the RQ area under the curve (AUC) was also lower in the SIT group (mean difference of −6.62, 95% CI −12.00 to −1.24; *p* = 0.0161). The contribution of fat to energy expenditure increased after SIT during the postprandial period whereas the contribution of carbohydrates decreased. The AUC for fat contribution was significantly higher (mean difference 2311.9, 95% confidence interval [CI] 578.8 to 4043.3; *p* = 0.0098) and the AUC for carbohydrate contribution was significantly lower (mean difference −2283.1, 95% CI −4040.2 to −527.1; *p* = 0.0117) in the SIT group than in the resting group. In conclusions, acute SIT might have a positive effect on metabolic responses to postprandial glucose and, accordingly, should be recommended for improving metabolism in people with overweight/obesity.

## Graphical abstract

There is evidence supporting that acute sprint interval training (SIT) might improve metabolic responses to postprandial glucose, but results are inconclusive. The present investigation indicates that acute SIT might have a positive effect on metabolic response to postprandial glucose and, therefore, it could be recommended for improving metabolism response in adults with overweight/obesity. The mechanism(s) involved in substrate utilization with SIT are not fully elucidated and the topic warrants further investigation.

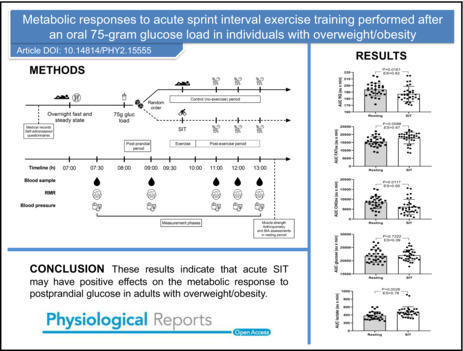

## 1 INTRODUCTION

Metabolic disorders are typically characterized by a constellation of clinical features including abdominal obesity, insulin resistance and/or impaired fasting glucose, and high blood pressure, which are known to increase the risk of developing type 2 diabetes mellitus (T2DM) and cardiovascular disease (CVD) (Ford, 2005). Although our understanding of the pathogenesis of metabolic disorders is incomplete, poor diet, smoking, excessive alcohol use, sedentary behavior, leisure‐time physical inactivity, and excess weight are all established risk factors (Yang et al., 2022). In Colombia, national examination surveys (in Spanish ENSIN) performed in 2005 to 2015 provided trend information on the distribution of obesity‐related risk factors in the general population. The rates of adult excess weight (overweight + obesity) were 46.0% (39.9% for men, 49.6% for women) in ENSIN 2005 and 56.5% (52.8% for men, 59.6% for women) in (ENSIN, 2015). Additionally, in a more recent cross‐sectional study on 890 healthy Colombian collegiate students (52% women), (Martínez‐Torres et al., 2017) reported that the prevalence of metabolic syndrome was 32.4%, which is very similar to the high prevalence rates of other countries with Hispanics/Latinos of diverse background (Heiss et al., 2014).

According to the carbohydrate–insulin model of obesity, the intake of high‐glycemic carbohydrates results in elevated postprandial insulin responses, which are believed to promote body fat accumulation, in turn increasing hunger and energy intake (Blundell & Macdiarmid, 1997). Impaired postprandial metabolism following meal ingestion, including postprandial hyperglycemia (PPG) and hyperinsulinemia, are important inter‐related risk factors for the development of CVD in patients with T2DM (Hiyoshi et al., 2017), and impaired PPG is associated with a pro‐inflammatory state and weakened endothelial function (Mah & Bruno, 2012). Regular exposure to periods of postprandial *activity* over the course of a day, including postprandial glycemia/insulinemia, places emphasis on the importance of this phase in the development of CVD, and also as a key prevention and treatment target.

Efforts to manage chronic diseases have led to attempts to change lifestyle behaviors, and physical exercise has been found to be one of the most important health‐promoting behaviors (Ramírez‐Vélez et al., 2017). Indeed, it is well established that regular exercise training plays a crucial role in both the treatment and prevention of many chronic diseases, in particular, in reducing the risk of CVD (Chen et al., 2022). Exercise may have added benefits if it is appropriately timed to the postprandial period (Erickson et al., 2017). For example, post‐meal exercise has been shown to trigger acute reductions in PPG (Derave et al., 2007; Khalafi et al., 2022). The effect of a single session of continuous aerobic training, such as walking and cycling, on fat (lipid) metabolism has also been investigated (Ho et al., 2011; Kolifa et al., 2004), yet most of the studies have been restricted to healthy, physical inactive, and/or normal‐weight individuals. In addition, exercise interventions tested have commonly been of 60–120 min duration at a moderate‐intensity ranging from 50% to 75% of maximum oxygen uptake (VO_2_max) (Herd et al., 2001; Ramírez‐Vélez et al., 2018).

Performing repeated bouts of high‐intensity “sprint”‐type exercise over several weeks or months induces profound changes in skeletal muscle (Blue et al., 2018). Sprint interval training (SIT) is defined as “all‐out” sprints (>90% of VO_2_max) interspersed with recovery periods. Regardless of the SIT protocol used, several studies have associated this type of exercise with a range of health benefits including large improvements in cardiorespiratory fitness, metabolic function, and body composition outcomes (Keating et al., 2017; Rosenblat et al., 2020). The effects of SIT on postprandial hyperinsulinemia have recently been meta‐analyzed by Khalafi et al., with the authors reporting mixed results (Khalafi et al., 2022). Acute SIT can reduce glucose and insulin levels in postprandial states in healthy participants (Au et al., 2021; Ferreira et al., 2011), in people with overweight and obesity (Little et al., 2014; Sargeant et al., 2021), and in patients with metabolic disorders (Freese et al., 2015; Metcalfe et al., 2018). These results overall suggest a potential beneficial role of acute SIT in improving metabolic functions, possibly due to the influence that this type of exercise has on the utilization of substrates*––*as fats and carbohydrates are oxidized simultaneously as energy sources (Atakan et al., 2022).

Few studies have investigated the acute effect of exercise on people with overweight/obesity, a group that includes the majority of the Colombian adult population, owing to their sedentary behavior and poor health and diet (Ferrari et al., 2022; García et al., 2022). The need for more specific recommendations for at‐risk individuals prompted the present randomized crossover trial, which aimed which. Therefore, the aim of this study was to explore the effects of acute SIT on metabolic response and substrate utilization after an oral 75‐gram glucose load in people with overweight/obesity. We hypothesized that acute SIT would improve postprandial metabolic responses.

## 2 MATERIALS AND METHODS

### 2.1 Participants

Thirty‐three participants with overweight or obesity (mean and standard deviation age 32.7 ± 8.3 years; 24 males, 9 females; body mass index 28.6 ± 4.4 kg/m^2^) participated in the study. All participants were initially screened by means of a medical questionnaire and physical examination, and those with metabolic, respiratory, or cardiovascular disorders, or any other major disease that limits the ability to perform the necessary exercises were excluded. All participants who had normal blood chemistry, were normotensive (blood pressure [BP] <140/90) and were not under medication. All participants performed activities of daily living and recreation but did not routinely participate in moderate‐ to high‐intensity aerobic exercise, and none had participated in resistance training in the last 3 months. The prevalence of self‐reported tobacco was 6%, and 18% of participants met the recommendation of 150 min per week. Participants' physical characteristics are shown in Table 1.

**TABLE 1:** Participant s' characteristics and fasting measurements (*n* = 33)

Characteristics	
Anthropometric and body composition parameters	
Sex (men/women), *n* (%)	24 (73%) 9 (17%)
Age, years	32.76 (8.33)
Body mass, kg	86.32 (14.61)
Height, m	1.74 (0.09)
Body mass index, kg/m^2^	28.62 (4.42)
Body fat, %	32.32 (6.81)
Skeletal muscle mass, kg	59.12 (10.96)
Metabolic and clinical parameters	
Resting energy expenditure, kcal	1906.13 (271.54)
Respiratory quotient, au	0.79 (0.06)
Energy expenditure/body mass, kcal	22.37 (2.31)
Oxygen consumption (VO_2_), L/min	277.87 (39.82)
Carbon dioxide production (VCO_2_), L/min	218.47 (34.86)
Fat substrate utilization, %	73 (65–89)
Carbohydrate substrate utilization, %	27 (12–35)
Basal systolic pressure, mmHg	127.53 (8.55)
Basal diastolic pressure, mmHg	78.23 (6.89)
Resting heart rate, beats/min	86.91 (7.94)
Triglycerides, mg/dl	138.79 (73.44–173.85)
Cholesterol, mg/dl	198.09 (179.75–211.01)
Glucose, mg/dl	84.90 (8.24)
Lactate, mmol/L	1.41 (1.10–1.57)
Grip strength, kg	37.25 (12.60)
Lifestyle parameters	
Tobacco (≥10 cigarettes per week), *n* (%)	2 (6)
Alcohol (≥1 times per week), *n* (%)	10 (30)
Physical activity levels (≥150 min per week), *n* (%)	6 (18)
Self‐reported morbidity	
Anemia, *n* (%)	1 (3)
Chronic low back pain, *n* (%)	3 (9)
Food allergy, *n* (%)	1 (3)
Tendinopathy, *n* (%)	1 (3)

*Note*: Continuous variables are expressed as mean (SD) or as mean (25th‐75th percentile value) where applicable, categorical variables as frequency and percentage. Self‐administered questionnaires were distributed to 33 participants to obtain information about demographic characteristic, lifestyles, and morbid disorders confirmed by the clinical diagnosis by physician. The determination of the lifestyle parameters was measured using the self‐reported questionnaire. To physical activity (PA) participation in the previous week, participants were also asked whether the pattern of PA reported in the questionnaire was consistent with the previous 7 days. PA was categorized as follows: insufficient: no PA practice (<150 min/week), or sufficient PA: five or more days of moderate‐intensity PA and/or walking, in combination or alone, at least 30 min/day, accumulating a minimum of 150 min/week according to WHO recommendations. In addition, habits such as smoking cigarettes (not smoking and those who currently smoke ≥10 cigarettes per week) and alcohol consumption (drink less than 1 day per week or not alcohol intake) were evaluated.

### 2.2 Study design and setting

**FIGURE 1:**
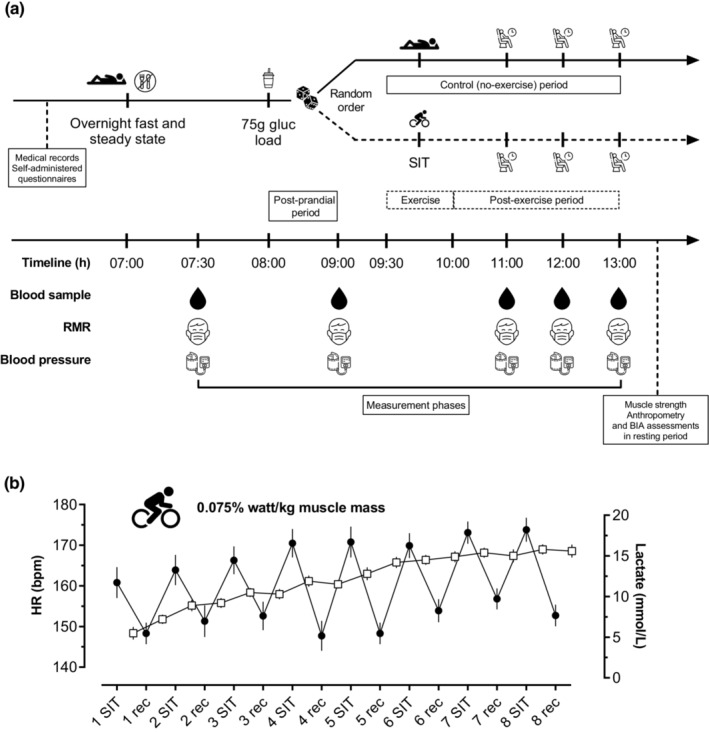
Overview of experimental protocol (a), heart rate, and lactate response from eight‐repeated 30‐s “all‐out” intervals (b). Study design (a). Face mask icons represents indirect calorimetry (metabolic cart) assessments. The soda cup icon represents the 75‐g oral glucose dose. The drop icons represent the capillary blood sample assessments. Other assessments were performed only on day 1 (not shown). The study protocol timeline is expressed as hours. BIA, Bioelectrical impedance analysis assessment. Muscle strength (grip strength), anthropometry, and BIA assessments were performed only on day 1. Heart rate (HR ●) and blood lactate (□) concentration relationships to eight‐repeated 30‐s “all‐out” response (b). Data represent mean (standard error). RMR, resting metabolic rate.

The participants performed two different trials in random order and separated by at least 5 days for the following purposes: (1) anthropometric and body composition measurements and completed in random order, no exercise (control, resting group) or (2) SIT performed on a cycle ergometer (Monark 894 E, Monark, Varberg, Sweden). Each trial began at 07:00 a.m. after an overnight fast (10–12 h). They were instructed to avoid caffeine, alcohol, strenuous, and exhaustive physical activity for 2 days before the exercise tests. For the no exercise (control, resting group) and the SIT trial, participants rested in a supine position for 30 min and then either ingested an oral carbohydrate liquid meal (Meal; partial hydrolysate of starch; 75 g glucose in a volume of 250 ml, this is the standard solution used for the oral glucose tolerance test; Dextrosol uva HYCEL; Grupo JAFS). The participants then returned to a supine position just after completing the oral glucose load (75 g) for 1 h. After experimental condition (control or SIT trial), participants remained in rest position 60,120, and 180 min after the ingestion of a 75‐g oral glucose challenge, (Figure 1a). During all periods, the room temperature was maintained at 24 ± 1°C by a thermal feedback device. This trial was performed from December 2020 to July 2021 at the University of Valle, Cali, Colombia.

### 2.3 Measurements

Body mass (kg) and height (m) measurements were determined using a balance scale (Seca 284™, SECA). Body mass index was calculated as weight/height^2^ (kg/m^2^). Body composition was assessed using tetrapolar bioimpedance analysis (BC‐418 MA; Tanita Corp.). Grip strength was measured with a Takei digital hand grip dynamometer using a standard protocol (Ramírez‐Vélez et al., 2021). For each measured period, heart rate, and systolic and diastolic brachial BP were evaluated with an OMRON (OMRON Healthcare., Tokyo, Japan) automated blood pressure monitor and results were recorded as the mean of two measurements. The commercially available biosensor Accutrend Plus® portable (Roche Diagnostic Australia Pty Ltd.) and the Lactate Pro 2 Blood Lactate Meter® portable were used to measure glucose, triglycerides, total cholesterol, and lactate by venous/capillary sampling, respectively. Metabolic data were collected using the COSMED Q‐NRG+® calorimeter, which included the following: carbon dioxide production (VCO_2_), oxygen consumption (VO_2_), resting energy expenditure (REE), respiratory quotient (RQ) (ratio between exhaled CO_2_ and inhaled O_2_), and the rate of substrate utilization (fat and carbohydrates). The first 5‐min of data were discarded, and the remaining 10 min were averaged. Carbohydrate utilization was estimated using Frayn's equation (Frayn, 1983). For analysis of resting metabolic rate (RMR), energy expenditure (EE), and carbohydrate utilization, the nitrogen urinary excretion was considered to be zero. The respiratory exchange ratio (RER; i.e., VCO_2_‐to‐VO_2_ ratio) was also calculated, and the abbreviated Weir equation (Weir, 1949) was used to estimate RMR and EE. In addition, self‐administered questionnaires were distributed for participants to obtain information about demographic characteristic, lifestyles, and morbid disorders confirmed by physician.

### 2.4 Sprint interval training protocol

Participants completed in random order, 5 days apart, no exercise (control, resting group) or SIT performed on a cycle ergometer (Monark 894 E, Monark, Varberg, Sweden). After a baseline measure, 12 min (4 min for SIT and 8 min for recovery interval) of exercise was performed 90‐min after an oral 75‐gram glucose load. Sessions consisted of eight‐repeated 30‐s “all‐out” efforts on a manual braked cycle ergometer against a resistance equivalent to 0.075 kg/kg muscle mass (i.e., a Wingate test). Participants were instructed to begin pedaling as fast as possible against the ergometer's inertial resistance, ∼2 s before the appropriate load was applied, and were verbally encouraged to continue pedaling as fast as possible throughout the 30‐s test. During the 1‐min recovery period between tests, they remained seated on the ergometer and either rested or were permitted to cycle at a low cadence (50 to 60 revolutions/rotations per minute) against a light resistance (~50 W) to reduce venous pooling in the lower extremities and minimize feelings of light‐headedness or nausea.

Prior to test, the maximal heart rate was calculated as 220 minus age. The target exercise intensity for each individual was 90%–95% of the estimated maximal heart rate. Heart rate was monitored continuously during cycling using a wireless chest‐strap monitor (Polar A300, Polar, Denmark). Each interval and resistance was varied as needed to maintain the heart rate within a range of ±5 beats/min of the target heart rate. As shown in Figure 1b, the mean (SD) heart rate was 169 (16) beats/min (defined as 90% of the predicted heart rate). The average blood lactate concentration during SIT (11.92 [3.24 mmol/L]) was ~10‐fold higher than the baseline level (1.41 [0.47 mmol/L]); all *p*'s < 0.01, Figure 1b.

### 2.5 Statistical analyses

Continuous variables are expressed as mean, least squares mean, standard deviation, standard error, or (25th–75th percentile value) where applicable, categorical variables as frequency and percentage. For each set of data, normal distribution was verified by a Shapiro–Wilk test. Differences in parameters between the resting versus SIT trial were analyzed for each time point using a two‐factor repeated‐measures ANOVA, and two‐sided Student's *t*‐tests were used to compare two sequences (resting vs. SIT trial). To compute the postprandial measured gas exchange response in a single outcome, we calculated the incremental area under the curve (AUC) using the trapezoidal rule (Wolever, 2004) minus the baseline (i.e., post‐prandial and 3‐trials post‐exercise period) value for each parameter (RQ, fat, and carbohydrate utilization), as well as for the glucose, lactate, and BP values. The level of significance for analyses was set at *p* < 0.05, and significant interactions and main effects were subsequently analyzed using Tukey's honestly significant difference post hoc test. To make inferences about magnitude of the effect for two sequences and AUC parameters, effect sizes (ESs) were calculated (Cohen, 1990) and expressed as 90% confidence limits (Batterham & Hopkins, 2006). The following formula was used to calculate the ES: [(post mean−pre mean)/SD_pooled_], where SD_pooled_ = √[(SD_pre_^2^ + SD_post_^2^)/2] (Cohen, 2013). An ES of <0.2 was considered to be trivial, 0.2–0.6 small, 0.6–1.2 moderate, 1.2–2.0 large, and 2.0–4.0 very large (Batterham & Hopkins, 2006). Statistical analyses were performed using GraphPad Prism version 9.00.

## 3 RESULTS

**FIGURE 2:**
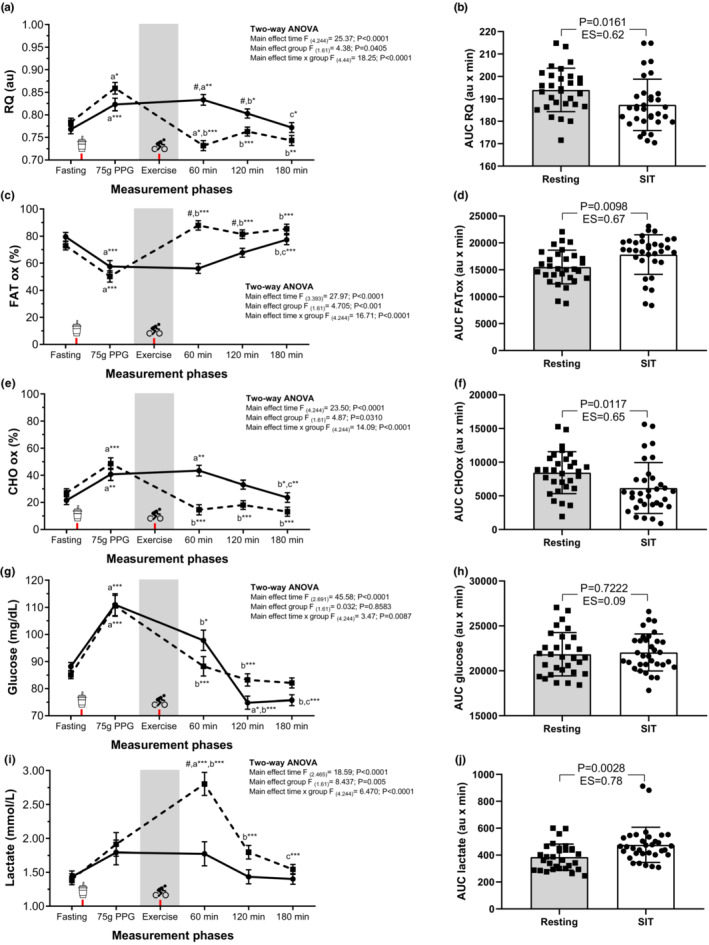
Effect of resting or SIT protocol on metabolic outcomes during the fasting, postprandial, and exercise periods. Respiratory quotient (RQ, a), RQ total response [area under the curve (AUC)] for each group (b), Fat utilization (c), Fat total response [AUC)] for each group (d), Carbohydrate (CHO) utilization (e), CHO total response [AUC)] for each group (f), capillary blood glucose concentrations (g), capillary blood glucose concentrations total response [AUC)] for each group (h), capillary lactate concentrations (i), capillary lactate concentrations total response [AUC)] for each group (j). Fasting values correspond to the resting metabolic rate period, that is, before the 75‐g glucose load, while 60, 120, and 180 represent the time in minutes for gas exchange data after the 75‐g glucose intake. The soda cup icon (x‐axis) represents the moment in which the glucose (75‐g dose, postprandial glycemia PPG) was provided. The bike icon (x‐axis) represents the moment in which the exercise was started. All AUCs are presented mean (SD) except on the figures where data are plotted as least squares mean (SEM) for better clarity. a Differences with fasting; b differences with 75‐g dose PPG; and c differences with 60 min. ***p < 0.0001; **p < 0.001; *p < 0.05. # Between group differences, *p* < 0.001, using Student's two‐sided tests. Resting (solid line) or SIT (dashed line) groups.

After an oral 75‐gram glucose challenge (60 min), the RQ increased from baseline and trial sequences (time effect *p* < 0.0001; group effect *p* = 0.0045; and time × group interaction effect *p* < 0.0001). However, at 120‐min after exercise, the least squares mean RQ was significantly lower in the SIT group (0.76 [0.01]; *p* < 0.0001) than in the resting group (0.80 [0.01]; *p* = 0.036), with a least squares mean difference of −0.04 (0.01); *p* = 0.0128 (Figure 2a). Likewise, the RQ AUC was lower in the SIT group than in the resting group (mean difference of −6.62, CI 95% −12.00 to −1.24; *p* = 0.0161, Figure 2b). The calculated ES was moderate (ES = 0.62) for SIT.

The effect of SIT versus resting on substrate contributions was significant (*p* < 0.05). The fat contribution to energy expenditure significantly increased in the SIT group at postexercise, both at 60 min and 120 min (group effect; *p* < 0.0001, time × group interaction effect; *p* < 0.0001; Figure 2c). Moreover, the fat utilization AUC was significantly higher in the SIT group than in the resting group (mean difference 2311.9, 95% confidence interval [CI] 578.8 to 4043.3; *p* = 0.0098; Figure 2d), with ES moderate (ES = 0.67) for SIT. The carbohydrate contribution to energy expenditure was also significantly different between the groups (group effect; F = 4.87, *p* = 0.0310; time × group interaction effect; F = 14.09, *p* < 0.0001). The carbohydrate utilization AUC was significantly lower in the SIT group than in the resting group (mean difference −2283.1, CI 95% −4040.2 to −527.1; *p* = 0.0117; Figure 2f), with ES moderate (ES = 0.65) for SIT.

The glucose time‐course responses are shown in Figure 2g. The least squares mean for glucose was lower in the 60‐min SIT group than in the resting group (88.26 [3.74] vs. 97.80 [3.57]). No significant differences were found between the two trials (group effect F = 0.032; *p* = 0.8583) or for the AUC (*p* = 0.7222) (Figure 2h); however, a time effect (F = 45.58; *p* < 0.0001) and time × group interaction (F = 3.47; *p* = 0.0087) was found. Contrastingly, lactate was significantly higher in the SIT group than in the resting group at 60‐min postexercise (1.77 [0.17] vs. 2.80 [0.17]; time effect F = 18.59; *p* < 0.0001, time group F = 8.43; *p* = 0.0005; and time × group interaction effect F = 6.47: *p* < 0.0001; Figure 2i). As expected, the lactate concentration AUC was significantly higher in the SIT group than in the resting group (*p* = 0.0028; Figure 2j), with ES moderate (ES = 0.78) for SIT.

**FIGURE 3:**
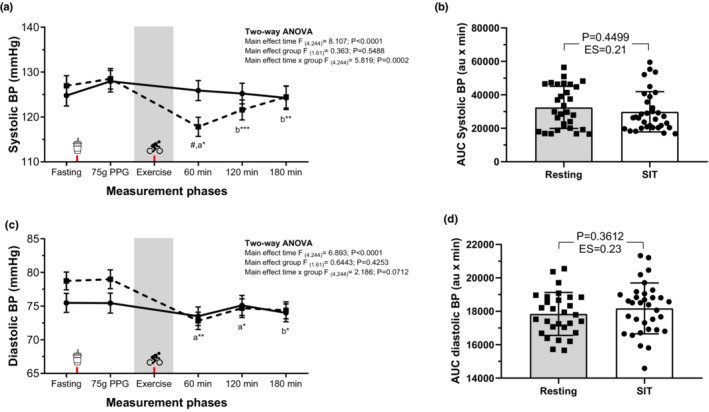
Effect of resting or SIT trial on brachial blood pressure during the fasting, postprandial, and exercise period. Systolic blood pressure (BP, a), Systolic BP total response [area under the curve (AUC)] for each group (b), Diastolic BP (c), Diastolic BP total response [AUC)] for each group (d). Fasting values correspond to the resting metabolic rate period, that is, before the 75‐g glucose load, while 60, 120, and 180 represent the time in minutes for gas exchange data after the 75‐g glucose load. The soda cup icon (x‐axis) represents the moment in which the glucose (75‐g dose, postprandial glycemia PPG) was provided. The bike icon (x‐axis) represents the moment in which the exercise was provided. All AUCs are presented mean (SD) except on the figures where data are plotted as least squares mean (SEM) for better clarity. ^a^ Differences with fasting; and b differences with 75‐g dose PPG.‐ ‐‐‐ ***p < 0.0001; **p < 0.001; *p < 0.05. # Between group differences, *p* < 0.001, using Student's two‐sided tests. Resting (solid line) or SIT (dashed line) groups.

A significant decrease in both systolic and diastolic BP was observed (time effect *p* < 0.0001) at 60, 120, and 180 min of the postprandial period in the SIT group, with no differences between the groups (group effect; *p* > 0.05); (Figure 3a and Figure 3c); however, there was no significant change in the total response for BP AUC (Figure 3b and Figure 3d). The ES was trivial for both systolic (ES = 0.21) and diastolic BP (ES = 0.23).

## 4 DISCUSSION

In the present study, we aimed to explore the effects of acute SIT on metabolic response and substrate utilization after an oral 75‐gram glucose load in individuals with overweight/obesity. We found that the fat contribution to energy expenditure and lactate levels significantly increased during the postprandial period after SIT, whereas the carbohydrate contribution was reduced. Moreover, the fat contribution and lactate concentration AUC were significantly higher in the SIT group than in the resting group, whereas the carbohydrate contribution AUC was lower. Furthermore, the RQ was lower in the SIT group than in the resting group at 120 min of the postprandial period and the RQ AUC was also lower in the SIT group. These results indicate that acute SIT may have positive effects on the metabolic response to postprandial glucose in adults with overweight/obesity.

Exercise is well‐documented to increase fat oxidation, which is principally due to the decrease of re‐esterification (Ho et al., 2011; Iwayama et al., 2015; Maunder et al., 2018; Petitt & Cureton, 2003; Trombold et al., 2013). Our findings confirm past observations reporting that a clear determinant of body fat oxidation rate is exercise intensity reviewed in (Purdom et al., 2018), and we show that acute SIT leads to a greater increase in fat oxidation rate at 60 min and 120 min postprandial. In accord with our findings, Yang et al. reported that high‐intensity exercise could induce an increase in postprandial fat oxidation rate in healthy males (Yang et al., 2018). A recent study also indicated that fat oxidation is significantly higher after Tabata exercise (a type of high‐intensity exercise consisting in eight rounds of 20 s of exercise and 10 s of rest) after a high‐fat meal in eleven healthy males (Pearson et al., 2020a). Moreover, the impact of exercise on postprandial fat oxidation has been recently demonstrated in a meta‐analysis and systematic review (Pearson et al., 2020b). Taken together, the findings suggest that acute exercise training might have protective health benefits in adults with overweight/obesity, such as improved fat oxidation after a glucose load. Although the underlying mechanisms for this protective effect are not yet well understood, it has been proposed that fat oxidation in the muscle is increased due to the stimulation of lipolysis in adipose tissue, an augmented muscle blood flow and, possibly, enhanced translocation of the lipoprotein receptor CD36 into the cell membrane (Dyck et al., 1996; Muscella et al., 2020).

In addition to fats, it is known that carbohydrates are simultaneously oxidized during energy production (Spriet, 2014). In the present study, postprandial carbohydrate utilization was lower in the SIT group than in the resting group and the AUC carbohydrate contribution was also lower. It seems that acute exercise training might improve glycemic control, supporting the notion that it is a useful strategy for improving carbohydrate metabolism in inactive individuals with overweight/ obesity. Our results are in accord with a previous study conducted in healthy, normal weight young women with a sedentary lifestyle (Hashimoto et al., 2015), supporting that postprandial aerobic exercise is effective for the promotion of postprandial carbohydrate metabolism. By contrast, (Bittel et al., 2021) concluded that a single bout of resistance exercise had no effect on carbohydrate oxidation; however, it should be noted that this study examined the effect of resistance training in a cohort of obese men with prediabetes. Therefore, differences in exercise modality and health status are likely to be responsible for the discrepancy between results.

Regarding RQ, which reflects substrate utilization when energy is expended, we found that while this metric increased at 60 min after a glucose load, it was significantly lower in the SIT group than in the control group at 120 min postprandial. Our findings confirm prior observations of differential RQ responses to exercise training, with high‐intensity exercise shown induce a greater reduction in RQ (Burgomaster et al., 2005; Júdice et al., 2021). Nevertheless, a direct comparison with other studies is difficult due to differences in factors affecting RQ (diet, pre‐training meal, age, sex, or weather conditions) (Charrière et al., 2016). Moreover, our study, in agreement with prior evidence (de Lima et al., 2015; Gordon et al., 2021), found that lactate levels significantly increased after SIT during the postprandial period.

In the last decades the consumption of sugars in food and beverages has risen alarmingly and this is known to be associated with increased risk factors for CVD including dyslipidemia, hypertension, diabetes, or non‐alcoholic fatty liver diseases (Stanhope, 2016). Thus, effective strategies such as SIT should be promoted in healthy adults with overweight/obesity to improve metabolic responses to postprandial glucose (Khalafi et al., 2022; Solomon et al., 2020).

The present study has some limitations that should be acknowledged. First, the study population comprised healthy overweight and obese adults that did not routinely participate in moderate‐ to high‐intensity aerobic exercise, and none had participated in resistance training in the last 3 month. As training status, sex and nutritional status of the individuals are determinants of metabolic response, our results may not be generalizable to other populations with different characteristics. Accordingly, these determining factors must be considered when interpreting results between studies. Second, the small sample size might be considered as a potential limitation, and future studies in larger cohorts including individuals with different characteristics (i.e., normal weight or physically active) are needed to further elucidate how acute SIT and metabolic responses are related. Third limitation is that we did not control the pre‐test nutritional habits of the participants, which could have affected metabolic responses. Future studies should examine whether diets high or low in carbohydrates or fats have a relevant effect on total oxidation rates of the substrates (Peric et al., 2016). Fourth limitation is that we did not examine other metabolic/hormonal factors such as antioxidant status, insulin/incretins, IL‐6 and/or TNF‐α levels, that typically mitigates postprandial glucose, and this should be studied in future research. Moreover, although our results might support a potential metabolic benefit of SIT, further studies comparing SIT and other types of aerobic exercise could be necessary to support these preliminary findings. Lastly limitation is related to bioelectrical impedance analysis assessments. Therefore, the bioelectrical impedance analysis devices (BC‐418 MA; Tanita Corp., Tokyo, Japan) is appropriate to use for body composition assessment in a healthy adult population (Johnson et al., 2012). In this line, (Pineau et al., 2007) reported significantly correlated body fat percentage of bioelectrical impedance analysis versus ultrasound techniques (r = 0.91), result showed comparable correlations with those of (Johnson et al., 2012) (r = 0.862) respectively.

The main strength of our study is that, to our knowledge, this is the first to investigate the effect of acute SIT on metabolic responses and substrate utilization after glucose consumption in inactive adults with overweight/obesity from the Latin‐American population. In addition, we provide measurements of these postprandial responses at multiple time points to better describe their temporal course.

In conclusion, the present investigation indicates that acute SIT might have a positive effect on metabolic response to postprandial glucose and, therefore, it could be recommended for improving metabolism response in adults with overweight/obesity. The mechanism(s) involved in substrate utilization with SIT are not fully elucidated and the topic warrants further investigation.

## AUTHOR CONTRIBUTIONS

Hugo Alejandro Carrillo‐Arango, Mikel Izquierdo, and Robinson Ramírez‐Vélez conceived and designed study; Robinson Ramírez‐Vélez and Miguel Alejandro Atencio‐Osorio performed statistical analysis; Hugo Alejandro Carrillo‐Arango, Miguel Alejandro Atencio‐Osorio, and Robinson Ramírez‐Vélez experimental phase; all authors interpreted results of analysis; Carlos Alejandro López‐Álban and Edna J. Nava‐González prepared figures; Edna J. Nava‐González, María Correa‐Rodríguez, and Robinson Ramírez‐Vélez drafted manuscript; all authors edited and revised manuscript; all authors approved final version of manuscript.

## FUNDING INFORMATION

The EXERMET study was supported by Universidad del Valle. They do not have influence or authority about collection, management, analysis, and interpretation of data; writing of the report; and the decision to submit the report for publication.

## CONFLICT OF INTEREST

No conflicts of interest, financial or otherwise, are declared by the authors.

## ETHICS STATEMENT

Participants were fully informed of the risks of the study and signed informed consent forms before any procedure. This study was approved by The Universdad del Valle Ethics Committee (ID number: 174–020 and 018–020, Cali, Colombia) and complied with the Declaration of Helsinki.
